
JOURNAL INFORMATION
==============================
NLM Title Abbreviation: Eur J Gen Pract
Journal ID: Eur J Gen Pract
NLM Journal ID: 9513566

The European Journal of General Practice

ISSN: 1381-4788
EISSN: 1751-1402
Publisher: Taylor & Francis

ARTICLE INFORMATION
==============================
PMCID: PMC9980032
DOI: 10.1080/13814788.2023.2171394
Article ID: 2171394
Article version: 1
Article version: Version of Record
Subjects: Abstract, Abstracts

Optimising the organisation of family medicine practice. Selected abstracts from the 94th EGPRN conference, Istanbul, Turkey, 12–15 May 2022

All abstracts of the conference can be found at the EGPRN website https://www.egprn.org/page/conference-abstracts

Abstracts

European Journal of General Practice

Electronic publication date: 2023 Feb 27
Publication date: 2023
Volume: 29
Issue: 1
Electronic Location ID: 2171394
Copyright: © 2023 The Author(s). Published by Informa UK Limited, trading as Taylor & Francis Group.
Copyright year: 2023
Copyright holder: The Author(s)
License: This is an Open Access article distributed under the terms of the Creative Commons Attribution License (http://creativecommons.org/licenses/by/4.0/), which permits unrestricted use, distribution, and reproduction in any medium, provided the original work is properly cited.
License URL: https://creativecommons.org/licenses/by/4.0/

Figure count: 0
Table count: 0
Page count: 9

==============================

Subjects: Abstract, Abstract

 

Keywords: Organisation of care, COVID-19, prevention, clinical topics & research

Subjects: Abstract, Keynote Lectures

International keynote lecture: Assessing the quality of primary care delivery

Ketiš Zalika Klemenc
Community Health Cntre Ljubljana , Ljubljana, Slovenia
CONTACT zalika.klemenc@um.si

Subjects: Abstract, Keynote Lectures

National keynote lecture: Establishing primary care; Turkey and Azerbaijan primary care organization

Akman Mehmet
Marmara University School of Medicine, Department of Family Medicine , Istanbul , Turkey
CONTACT makman4@gmail.com

Subjects: Abstract, Poster Prize Winner

Very simple PDF-based online ageing game equivalent enhances medical students’ understanding of elderly patients

Geier Anne-Kathrin
Lippmann Stefan
Rau Antje
Schrimpf Anne
Bleckwenn Markus
Deutsch Tobias
Department of General Practice, Medical Faculty, University of Leipzig , Leipzig , Germany
CONTACT Annekathrin.geier@medizin.uni-leipzig.de
Keywords: Online learning, aging game, aging simulation, geriatric medicine, awareness

Subjects: Abstract, Selected Abstracts Organisation of Care

Higher patient waiting times are associated with higher chronic stress of general practice personnel: Results of the cluster-randomised IMPROVEjob study

Göbel Julian
Linden Karen
Grot Matthias
Werners Brigitte
Degen Lukas
Seifried-Dübon Tanja
Rind Esther
Eilerts Anna-Lisa
Pieper Claudia
Schröder Verena
Rieger Monika A.
Weltermann Birgitta
Institute of General Practice and Family Medicine , Bonn , Germany
CONTACT julian.goebel@ukbonn.de
Keywords: Waiting times, stress, general practitioners, practice personnel

Subjects: Abstract, Selected Abstracts

The desire to be a better doctor versus the lack of time and resources: Promotors and inhibitors for quality improvement work in general practice. A qualitative analysis of 2715 free-text replies from participants in a quality improvement project

Eide Torunn Bjerve
Øyane Nicolas
Høye Sigurd
Department of General Practice, University of Oslo , Oslo , Norway
CONTACT torunnbjerveeide@gmail.com
Keywords: Quality improvement, continuous medical education, antibiotic prescribing

Subjects: Abstract, Selected Abstracts

The process of diagnosis of cancer and the effect of the primary care in this process: A single-centre survey analysis

Dilbaz Ayşenur Duman
Çifçili Saliha Serap
Karadeniz Technical University , Trabzon , Turkey
CONTACT aysnr27dmn@gmail.com
Keywords: Cancer diagnosis, early diagnosis, cancer delay, diagnostic delay

Subjects: Abstract, Selected Abstracts

Developing a Serbian strategy to improve implementation of primary family violence care

Knežević Snežana
Van Hansewyck Nell
Đikanović Bosiljka
Bravo Raquel Gomez
Ak Filiz
Alonso Carmen Fernandez
Pas Lodewijk
University of Kragujevac & Health Center Kraljevo , Kraljevo , Serbia
CONTACT lesta59@yahoo.com
Keywords: Primary health care, family violence, barriers, facilitators, implementation

Subjects: Abstract, Selected Abstracts

Contextual factors associated with successful implementation of the evidence-based health promotion intervention Prescibe Vida Saludable

Rogers Heather L.
Harnando Susana Pablo
Nunez-Fernandez Silvia
Sanchez Alvaro
Martos Carlos
Moreno Maribel
Grandes Gonzalo
Biocruces Bizkaia Health Research Institute , Barakaldo , Spain
CONTACT rogersheatherl@gmail.com
Keywords: Health promotion, implementation science, primary care

Subjects: Abstract, Selected Abstracts

Effectiveness of combining patient follow-up with an educational intervention on self-management skills of type 2 diabetes mellitus patients: A primary care pragmatic trial

Aktemur Ayşenur
Kılıç Güldeniz
Doğrusever Handenur Tan
Çifçili Saliha Serap
Marmara University , Pendik , Turkey
CONTACT aysenursvl@gmail.com
Keywords: Type 2 diabetes mellitus, self-management, intervention, patient-empowerment

Subjects: Abstract, Covid-19

Quality and outcome of diabetes care during the COVID-19 pandemic in a primary care setting in Switzerland

Lüthi Benjamin Sebastian
Di Gangi Stefania
Hernandez Laura Diaz
Zeller Andreas
Zechmann Stefan
Fischer Roland
Centre for Primary Health Care, University of Basel , Basel , Switzerland
CONTACT benjamin.s.luethi@gmail.com
Keywords: Diabetes mellitus, primary care, COVID-19 pandemic, quality indicators, diabetes outcomes

Subjects: Abstract, Covid-19

COVID-19 severity prediction based on patient risk factors and number of vaccines received

Vinker Shlomo
Israel Ariel
Schäffer Alejandro A.
Merzon Eugene
Green Ilan
Magen Eli
GolanCohen Avivit
Ruppin Eytan
Leumit Health Services, Family Meidcine , Ashdod , Israel
CONTACT vinker01@gmail.com
Keywords: COVID-19, disease severity, calculator, diabetes, obesity, kidney disease

Subjects: Abstract, Covid-19

Acceptability and feasibility of self-organised blood sample collection for SARS-CoV-2 antibody screening in persons with a high risk for a severe COVID-19 disease progression

Schröder Dominik
Dopfer-Jablonka Alexandra
Klawonn Frank
Müller Frank
Heinemann Stephanie
Department of General Medicine, University Medical Center Goettingen , Germany , Göttingen , Germany
CONTACT dominik.schroeder@med.uni-goettingen.de
Keywords: SARS-CoV-2, acceptability, feasibility, blood sample

Subjects: Abstract; Prevention, Clinical Topics & Research

Pneumococcal vaccination coverage and adherence to recommended dosing schedules in adults: A repeated cross-sectional study in the INTEGO morbidity registry

Janssens Arne
Abels Chloé
Merckx Barbara
Abreu André Bento
Vaes Bert
KU Leuven , Leuven , Belgium
CONTACT arne.janssens@kuleuven.be
Keywords: General practice, adult pneumococcal vaccination, coverage rate, equity

Subjects: Abstract; Prevention, Clinical Topics & Research

The SPICES cardiovascular risk assessment in general population: Quantitative data and implantation clues

Goff Delphine Le
Perraud Gabriel
Aujoulat Paul
Derriennic Jérémy
Barais Marie
Reste Jean Yves Le
Faculté de médecine, Médecine Générale , Brest , France
CONTACT docteurdlegoff@gmail.com
Keywords: Cardiovascular disease prevention, general population, implementation

Subjects: Abstract; Prevention, Clinical Topics & Research

Quality of life and physical activity in prefrail individuals over 70 years in primary care

Castell-Alcala Victoria
Prieto-Aldana María
Gutiérrez-Misis Alicia
Viñals Rosa Julian
Schwarz Christine
Gálvez-Fernández Marta
Rodríguez-Barrientos Ricardo
Castro Elena Polentinos
SERMAS (SERVICIO MADRILEÑO DE SALUD). Prımary health care centre dr Castrovıejo , Madrid , Spain
CONTACT mariavictoria.castell@salud.madrid.org
Keywords: Pre-frailty, quality of life, exercise, aged, primary health care, sex

Subjects: Abstract; Prevention, Clinical Topics & Research

Orthopaedic corticosteroid injection and risk of acute coronary syndrome: A case-control study

Thomas Katharine
Schonmann Yochai
Tel Aviv University Medical School, Department of Family Medicine , Tel Aviv , Israel
CONTACT marshykd@gmail.com
Keywords: Acute coronary syndrome, corticosteroid injections, intra-articular injections, orthopaedics, rheumatology

Subjects: Abstract; Prevention, Clinical Topics & Research

Towards de-identification of general practitioners’ electronic medical records for secondary research

Hauswaldt Johannes
Groh Roland
Zarei Alireza
Kaulke Knut
Schlegelmilch Falk
University Medical Center Göttingen, Institute of General Practice , Göttingen , Germany
CONTACT johannes.hauswaldt@med.unigoettingen.de
Keywords: Electronic medical record, secondary research, privacy protection, de-identification, health services research

Subjects: Abstract; Prevention, Clinical Topics & Research

Evaluation of drug use status, rational drug use level and interaction status between drugs of patients who have a chronic disease and apply to the education family health centre (FHC) to prescribe drugs

Göktaş Şems Azra Kaya
Göktaş Mücahit
Arica Seçil
Cemil Taşçıoğlu City Hospital , İstanbul , Turkey
CONTACT semskaya0393@gmail.com
Keywords: Chronic disease, rational drug use, drug

